# SEOM Clinical Guideline for gastrointestinal sarcomas (GIST) (2016)

**DOI:** 10.1007/s12094-016-1579-9

**Published:** 2016-11-28

**Authors:** A. Poveda, V. Martinez, C. Serrano, I. Sevilla, M. J. Lecumberri, R. D. de Beveridge, A. Estival, D. Vicente, J. Rubió, J. Martin-Broto

**Affiliations:** 1 Department of Medical Oncology, Instituto Valenciano de Oncología, IVO, Valencia, Spain; 2 Department of Medical Oncology, Hospital Universitario La Paz, Madrid, Spain; 3 Department of Medical Oncology, Vall d’Hebron Institute of Oncology, Hospital Universitario Vall d’Hebron, Barcelona, Spain; 4 Investigación Clínica y Traslacional en Cáncer, Instituto de Investigaciones Biomédicas de Málaga (IBIMA), Hospitales Universitarios Regional y Virgen de la Victoria de Málaga, Málaga, Spain; 5 Department of Medical Oncology, Complejo Hospitalario de Navarra, Navarre, Spain; 6 Musculoskeletal and Gastric Tumor Unit, Department of Medical Oncology, Hospital Universitario y Politécnico La Fe, Valencia, Spain; 7 Department of Medical Oncology, Instituto Catalán de Oncología, ICO-Badalona, Hospital Germans Trias i Pujol, Badalona, Spain; 8 Department of Medical Oncology, Hospital Universitario Virgen Macarena, Badalona, Spain; 9 Department of Medical Oncology, Instituto Catalán de Oncología, ICO-Girona, Hospital Josep Trueta, Girona, Spain; 10 Department of Medical Oncology and Insituto de Biomedicina, IBIS, Hospital Universitario Virgen del Rocio, Av. Manuel Siurot, s/n, 41013 Sevilla, Spain

**Keywords:** GIST, Imatinib, Sunitinib, Regorafenib

## Abstract

Gastrointestinal stromal tumors (GISTs) are the most common mesenchymal neoplasms of the digestive tract, and this disease has served as a paradigmatic model for successful rational development of targeted therapies. The introduction of tyrosine kinase inhibitors with activity against KIT/PDGFRA in both localized and advanced stages has remarkably improved the survival in a disease formerly deemed resistant to all systemic therapies. The Spanish Society of Medical Oncology (SEOM) guidelines provide a multidisciplinary and updated consensus for the diagnosis and treatment of GIST patients. We strongly encourage that the managing of these patients should be performed within multidisciplinary teams in reference centers.

## Prologue

Gastrointestinal stromal tumor (GIST) is the most frequent mesenchymal tumor of digestive tract but also the most frequent sarcoma with an average incidence of 12–14 cases per million inhabitants each year. With the emergence of tyrosine kinase inhibitors (TKI) as imatinib, this entity was redefined supported by consistent histologic background, kit immunostaining, and specific mutational profile. Since then, the clinical and basic research has increased the knowledge around GIST allowing the registry of three different lines of targeted therapies in advanced disease and demonstrating the role of adjuvant imatinib in localized high-risk GIST.

Network connection is necessary to offer the best prognostic information and therapeutic option for GIST patients. Genotype and multidisciplinary approach should be mandatory in the context of GIST. The current update of SEOM GIST guidelines points out on the standard diagnostic and therapeutic procedures. We invite you to consider a good compliance to these guidelines, which are an updated version of the previous [1], as well as to spread this information in your area of influence.

## Methodology

Spanish Society of Clinical Oncology (SEOM) and Spanish Group for Research on Sarcoma (GEIS) jointly convened an expert panel. This panel was in charge of systematic review of the literature, and each member is responsible of giving feedback of the entire document. Task of writing the manuscript and giving recommendations following ASCO evidence levels and recommendation grades (Table 1) was distributed accordingly. Therefore, expert consensus was based on clinical evidence and literature available at the time they are written.

**Table 1:** Levels of evidence and recommendation grades from American Society of Clinical Oncology (ASCO)

Levels of evidence
I. Evidence from meta-analysis focusing on well-designed and controlled trials. Randomized trials with low incidence of false negatives or positives
II. Evidence from at least one well-designed experimental study. Randomized trials with high incidence of false negatives or positives
III. Evidence from well designed but no randomized trial: phase I/II trials, cohorts, and case–control study
IV. Evidence from non-experimental trials as observational studies
V. Evidence from cases and clinical examples
Recommendation grades
A. There is a type I evidence or consistent findings in multiple studies with evidence II, III, or IV
B. There is a type II, III, or IV with mostly consistent findings
C. There is a type II, III, or IV with mostly inconsistent findings
D. There is scarce or no systematic empiric evidence

## Diagnostic evaluation

### Radiology

CT scan is the most common imaging technique for the diagnosis, initial evaluation of tumor extension, and post-treatment follow-up of GIST [2]. Contrast-enhanced CT scan with image acquisitions of the arterial and portal phases is indicated for evaluating tumor extension. The study of the liver parenchyma during the arterial phase is important, because any existing small liver metastases can be detected which may not be visible during the portal phase [3]. For follow-up purposes, non-contrast and portal phase CT should be enough. Recommendation: Choi [4], instead of RECIST, is the recommended criteria for radiological assessment [III, B] (Table 2). Quantification of median tumor density is measured through ROI, including the maximal tumor areas on images acquired in portal phase, and is expressed in Hounsfield units (UH).

**Table 2:** RECIST and Choi radiologic assessment criteria

Response	RECIST	CHOI criteria
Complete response (CR)	All lesions must disappear	All lesions must disappear No new lesions
Partial response (PR)	Decreasing size 30% of sum of target lesions	Decreasing size >10% or decreasing density ≥15% HU
Stable disease (SD)	Between PR and PD	Does not fulfill CR, PR or PD criteria No symptom deterioration due to tumor progression
Progressive disease (PD)	Target lesions increase >20%	Sum of longest diameters increases >10% without density decreasing New intratumoral nodules Increase in size or previous intratumoral nodules

**Fig. 1:**
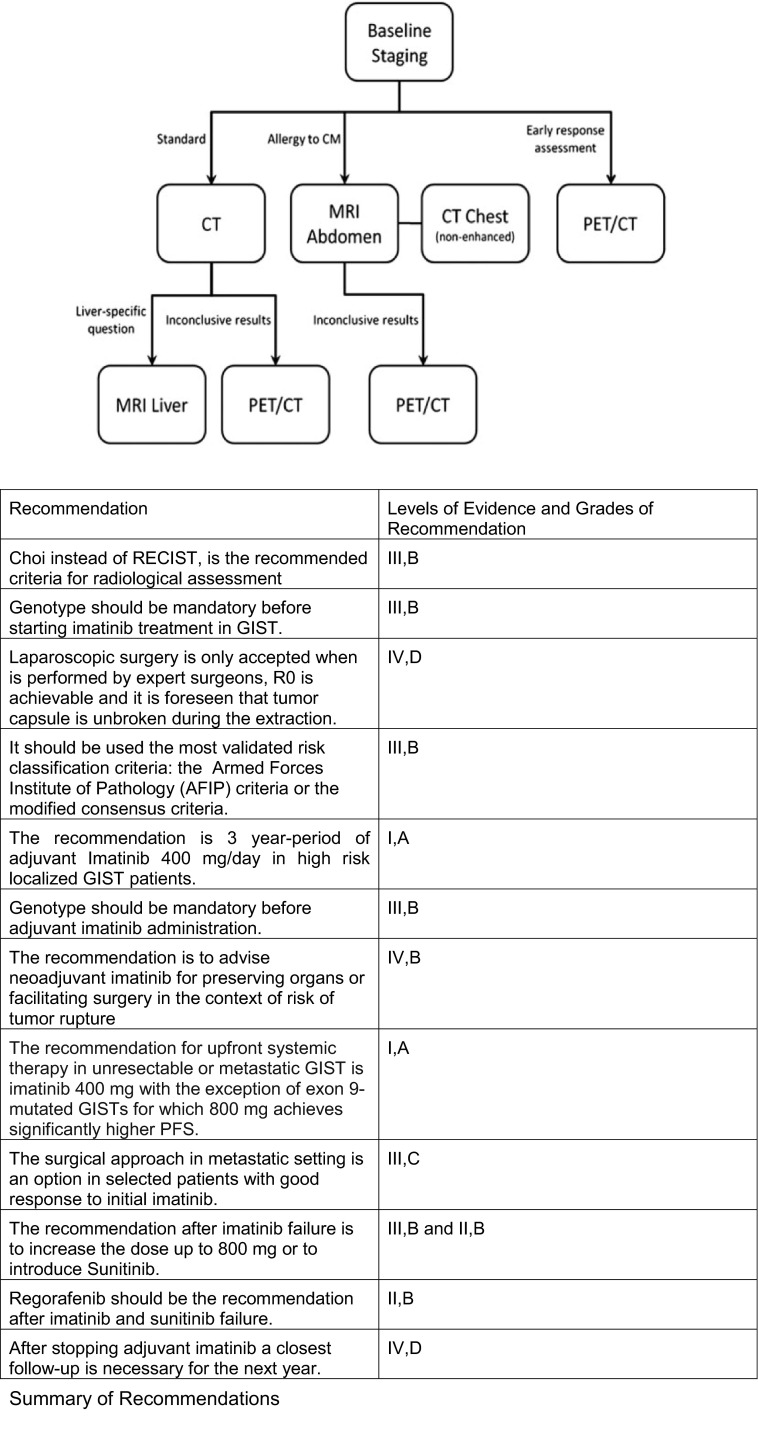
Algorithm of imaging techniques in GIST

Magnetic resonance imaging (MRI) is useful for the local study of tumors located in the pelvic area, in cases of potential resection of liver metastases due to the higher sensitivity in detecting small liver lesions and, moreover, is an alternative method to CT if contraindications to CT exist (Fig. 1). PET is reserved for inconclusive cases by other techniques, such as CT or MRI, or the early assessment of response to imatinib. FDG-PET can also be used to identify early resistance to treatment in patients, so that they can begin an alternative treatment.

### Histology

There is not a general consensus on the need of a preoperative histological diagnosis in the context of resectable, intramural, and clinical meaningful tumors of gastrointestinal tract. However, when neoadjuvant imatinib is considered for a downstaging manoeuvre, a CT-guided percutaneous core-biopsy (with sufficient material for an adequate mitotic count and molecular analysis) must be performed. In this context, it is necessary to both, a risk group classification and a genotype characterization to make correct therapeutic decisions.

*Macroscopic characteristics* GIST is rarely invasive, but sometimes, ulceration of the mucous membrane is observed [5]. GIST presents usually as solitary but in familial and Carney Triad, or in NF1-related GIST, multiple tumors can be seen. The pathology report must always include three-dimensional tumor measurement, and the existence of quantification of necrosis and distance between lesion and margin as incomplete resection is associated with poor prognosis. Likewise, information about the integrity of the tumor is relevant, since disruption of pseudocapsule is deleterious [6].

*Microscopic characteristics* Three histological types can be distinguished according to the cellular appearance: spindle cells (77%), *epithelioid* cells (8%), and *mixed* (15%). Importantly, mitosis counting has to be carried out in the most active areas. Although traditionally expressed as number of mitoses per 50 high-power fields (HPF), it is advisable to count mitosis in areas of 5 mm2, equivalent to 25 HPF with a 20X lens, or 21 HPF with a 22X lens [7] (this corresponds to 50 HPF in Miettinen risk classification).

*Immunohistochemistry* Over 95% of GIST have CD117 (c-kit) expression with diffuse cytoplasmic staining pattern but also rarely in the membrane or Golgi apparatus. Moreover, 70–90% also express CD34, 20–30% actin, 8–10% S-100, and desmin in 2–4%. DOG1 can optionally be included in the initial panel and is highly recommended in negative c-kit GIST-like tumors [8]. SDHB and SDHA are useful for identifying patients with *KIT/PDGFRA* wild type with SDH deficiency. SDHA negative immunostaining identifies those patients carrying mutation in the *SDHA* gene.

### Molecular biology

Gastrointestinal stromal tumors (GIST) characteristically harbor in 85% of cases activating mutations in KIT or PDGFRA genes which encode a tyrosine kinase receptor. These mutations are mutually exclusively. The most frequent mutation (70–75%) is located in the exon 11 juxta-membrane domain, followed by exon 9 mutation (extracellular domain) [9]. Less frequently, primary mutations in the adenosine triphosphate (ATP)-binding pocket (exon 13) or activation loop (exon 17) are found [10].

PDGFRA-activating mutations occur in 5–7% of GIST and they also encode a tyrosine kinase receptor (tyrosine kinase platelet-derived growth factor receptor alpha) [11]. Primary PDGFRA mutations could be found in the activation loop (6% cases), encoded by exon 18 (being the D842V, the most frequent mutation), the juxta-membrane domain (0.7%) encoded by exon 12, and the first tyrosine kinase domain (exon 14).

Finally, there is a subset of 12–15% of adult GISTs (90% of pediatric GISTs) which lack mutations in KIT and PDGFRA and are, therefore, called “wild type” GISTs [10]. In this subset, patients with BRAF mutations or succinate dehydrogenase (SDH) deficiencies can be found.

Nearly, half of the SDH-deficient patients have SDH subunit gene mutations, most commonly A (30%), and B, C, or D (together 20%). In the other 50%, epigenetic silencing (due to methylation) of the SDH complex seems to be the possible pathogenesis. There are two syndromes related to SDH-deficient GISTs: Carney Triad (characterized by multifocal gastric GIST, extra-adrenal and functional paraganglioma, and pulmonary chondroma) and Carney–Stratakis syndrome (GIST and paraganglioma), this latter with germline mutations. Neurofibromatosis-1-associated GISTs are also KIT and PDGFRA wild type, but not SDH deficient.

The most common mechanism involving TKIs resistance entails expansion of tumor clones harboring a range of secondary mutations in KIT or PDGFRA which are resistant to imatinib.

#### Mutational status clinical implications

Recommendation: Genotype should be mandatory before starting imatinib treatment in GIST [III, B]. Prognostic value in localized disease that could have therapeutic adjuvant implications [12] or predictive value that is especially relevant in neoadjuvant or metastatic scenario [13]. Cases harboring exon 11 are the most sensitive to imatinib. Patients with nine mutants have significantly better PFS if they were treated with 800 mg instead of 400 mg. In addition, D842V mutation in exon 18 of PDGFRA exhibits resistance to all the TKI registered in GIST.

## Localized disease management

### Surgery

The gold standard treatment for localized GIST is a complete removal achieving a R0-type surgery without tumor rupture. To gain this aim, the tumor should be radiologically resectable and the surgical morbimortality ought to be acceptable. In special cases with large tumors or complicated locations (e.g., rectum, gastroesophageal junction), the best treatment option should be discussed in a multidisciplinary context, because the unaffected tissue excision is not desirable and it is recommended to avoid multi-visceral resection or functional damages, being the neoadjuvant treatment a good tool in these conditions. Routinely, lymphadenectomy is not recommended [14].

Regarding margin resection, if the surgery was R1, re-excision could be offered, always sharing the decision with the patient and preventing loss of functionality. In low-risk tumors, there is no clear evidence that R1 margins imply a worse prognosis and wait and see could be a proper approach [15]. Recommendation: Laparoscopic surgery is only accepted when is performed by expert surgeons, R0 is achievable, and it is foreseen that tumor capsule is unbroken during the extraction [IV, D].

### Prognostic factors in localized resected gist

Relapse-risk assessment for surgically resected primary GIST is critical not only to provide prognostic information, but also to estimate the potential benefit of adjuvant imatinib. Prognostic factors in GIST include mitotic count (expressed as the number of mitoses on a total area of 5 mm^2^, tumor size and tumor site (extra-gastric location entails worse outcome). Spontaneous or intraoperative capsule rupture should also be recorded and considered as a very poor prognostic factor. Several risk-stratification systems have been proposed and include some or all the aforementioned prognostic factors. Recommendation: It should be used the most validated risk classification criteria: the Armed Forces Institute of Pathology (AFIP) criteria (Table 3) [7] or the modified consensus criteria [III, B]. AFIP risk criteria proved to be the fittest distinguishing low-, moderate-, and high-risk GIST patients in a GEIS series.

**Table 3:** Risk group classification according to AFIP criteria

Size (cm)	Mitotic count (50 HPF)	Location
Very low risk		
2–5	≤5	Gastric
Low risk		
>5 and ≤10	≤5	Gastric
2–5	≤5	Intestinal
Intermediate risk		
>10	≤5	Gastric
>5 and ≤10	≤5	Intestinal
2–5	>5	Gastric
High risk		
2–5	>5	Intestinal
>10	≤5	Intestinal
>5 and ≤10	>5	Gastric
>10	>5	Gastric
>5 and ≤10	>5	Intestinal
>10	>5	Intestinal

Tumor rupture has the consideration of peritoneal micro-metastases

### Adjuvant treatment

Several key phase III randomized clinical trials have clarified the value of imatinib in the adjuvant setting. Data from study ACOSOG Z9001 established that 1 year of imatinib was superior to placebo in terms of relapse-free survival (RFS) for resected GIST greater than 3 cm. This benefit was strengthened with the results of the following studies: EORTC 62024/GEIS-10 of 0 vs 2 years of imatinib (including intermediate- and high-risk patients) and the SSGX-VIII/AIO study of 1 vs 3 years of imatinib (including only high-risk patients) [16, 17]. Furthermore, the last study obtained an increase of overall survival (OS) with 3 years of imatinib compared with 1 year in high-risk patients (in accordance with NIH modifications). In a 2016 update with median follow-up of 90 months, benefit was maintained, with 5 year RFS of 71% for 3 years vs 52% for 1 year and 5 year OS of 92 vs 85%, respectively (HR 0.60; 95% CI 0.37–0.97; *p* = 0.036) [18]. Therefore, the recommendation is 3-year period of adjuvant imatinib 400 mg/day in high-risk localized GIST patients [I, A].

Adjuvant treatment for low-risk patients is not indicated, and currently, there is not enough data to recommend adjuvant treatment in intermediate-risk patients. Recommendation: Genotype should be mandatory before adjuvant imatinib administration [III, B], since mutations involving 557/558 of exon 11 in *KIT* gene determine a relapse-free-survival risk similar to high risk in patients with gastric and intermediate-risk GIST [12]. In addition, it makes no sense to administer adjuvant imatinib in the context of D842V mutation.

### Neoadjuvant treatment

Advantages of the neoadjuvant approach include cytoreduction to facilitate an R0 resection, the potential for organ preservation and a less invasive surgical approach and finally a decrease in the hypervascularity of the tumor, which can lead to a decrease in the risk of intraoperative bleeding or tumor rupture. Typical locations for this approach include the rectum, the esophagus, gastroesophageal junction, lesser curvature of stomach, and in tumors with a high risk of rupture. Maximal tumor response is seen usually after 4–12 months of treatment. Genotype is mandatory as it was explained before. Imatinib can be stopped the day before surgery and restarted as soon as the enteral route has been reestablished. Adjuvant treatment with imatinib should be given after surgery for a period of 3 years in total, both including the pre- and post-operative treatments. Therefore, the recommendation is to advise neoadjuvant imatinib for preserving organs or facilitating surgery in the context of risk of tumor rupture [IV, B].

## Advanced disease management

### Treatment of unresectable or metastatic disease

Imatinib mesylate (STI571, Gleevec^®^) is a selective tyrosine kinase inhibitor (TKI) of ABL, BCR-ABL, KIT, and PDGFR. The standard dose of Imatinib is 400 mg/day, and it was established based on two randomized phase III trials, in patients with c-kit positive metastatic or unresectable GIST, comparing daily doses of 400 vs 800 mg [19, 20]. The clinical benefit rates (CR + PR + SD) for 800 and 400 mg were 90 and 88% in NASG-S0033 study and 91 and 87%, respectively, in the EORTC one. The median PFS for patients treated with imatinib is around 22 months. Furthermore, there were no differences in overall survival and the toxicity profile was favorable in the 400 mg/d arm. A small but significant PFS advantage was documented for the high-dose arm in the EORTC trial. The most common adverse events with imatinib are edema (70%) (especially periorbital), nausea (50%), diarrhea (45%), myalgia (40%), fatigue (35%), dermatitis or erythema (30%), headache (25%), and abdominal pain (25%). The recommendation for upfront systemic therapy in unresectable or metastatic GIST is imatinib 400 mg with the exception of exon 9-mutated GISTs for which 800 mg achieves significantly higher PFS [I, A]. It is doubtful that imatinib should be recommended in *KIT/PDGFRA* wild type.

### Surgery in the context of metastatic disease

Several retrospective studies have demonstrated survival benefit of cytoreductive surgery and complete excision of residual metastatic disease following response to initial imatinib treatment, but it has never been demonstrated prospectively [21].

In the largest of these studies, 12-month progression-free survival and overall survival were 80 and 95%, respectively, but it is impossible to assess the specific contribution of surgery to the survival rates.

At the present time, the surgical approach in metastatic setting is an option in selected patients with good response to initial imatinib [III, C]. It is necessary to continue imatinib after the excision of all visible lesions to maintain disease remission, based on the evidence that imatinib interruption in metastatic disease results in rapid progression [II, A] [22]. For patients with limited disease progression, surgical debulking has been associated with a progression-free interval in the same range as for second-line treatment with sunitinib. Therefore, this may be a palliative option in the individual patient with limited progression while continuing imatinib [V, C].

### Systemic treatment following imatinib failure

While the majority of GIST patients respond to imatinib treatment, approximately 10–15% of them show primary resistance with a further 40–50% developing secondary resistance to the agent with a median time to progression of about 24 months. All clinical data, including lesion density on CT, potential drug interactions, and patient compliance to treatment, should be assessed prior to dose escalation of imatinib or switching to sunitinib.

When disease progresses at the dose of 400 mg/day, an increase to 800 mg/day is an option. Two studies (EORTC-ISG-AGITG and American Intergroup study S0033) have shown partial responses or stable disease for a certain period in about 30% of patients [19].

Sunitinib malate is an oral multitargeted inhibitor of KIT, PDGFRs, VEGFRs, and several other receptor tyrosine kinases. A pivotal phase III study reported that the response rate of imatinib-failure GIST to sunitinib was nearly 10%, and the clinical benefit rate was approximately 65% [23]. The median PFS of 6 months was more than four times longer than that of the placebo arm. On the basis in these results, sunitinib 50 mg/day on an intermittent dosing schedule of 4 weeks on treatment followed by 2 weeks off received multinational regulatory approval for the treatment of advanced imatinib-resistant or imatinib-intolerant GIST. Although no randomized trials to date have compared intermittent and continuous sunitinib dosing schedules, both are equally recommended. Asthenia, skin toxicity, diarrhea, hypertension, and hypothyroidism are the most frequent adverse events with sunitinib. Close monitoring of hypertension, cardiac function, and thyroid hormones is indicated during sunitinib therapy. The recommendation after imatinib failure is to increase the dose up to 800 mg [III, B] or to introduce Sunitinib [II, B].

### Resistance to imatinib and sunitinib

Regorafenib, an orally available multikinase inhibitor with activity against KIT, has recently been approved for the treatment of unresectable and/or metastatic GIST patients after failure of imatinib and sunitinib. A phase III randomized trial evaluated 28-day cycles of regorafenib 160 mg daily, 3 weeks on, 1 week off, using placebo as the comparator arm. Regorafenib treatment achieved an mPFS of 4.8 months, a CBR at 12 weeks of 52.6%, and an ORR of 4.5%. The toxicity profile of regorafenib was consistent with that of other kinase inhibitors with similar target spectrum, and the most common adverse events were hypertension, hand–foot skin reaction, and diarrhea [24]. Participation in clinical trials should be considered after regorafenib failure, since no standard treatment options are approved at this stage. Other therapeutic approaches might include imatinib rechallenge or pazopanib. Therefore, regorafenib should be the recommendation after imatinib and sunitinib failure [II, B].

## Follow-up

There are no clinical trials assessing follow-up of patients with GIST, and then, these recommendations are based on expert opinions. Follow-up recommendations are based on the risk of relapse, which depend on tumor localization, size, mitosis, and tumor rupture for localized and resected GIST. The aim of follow-up in GIST is to detect subclinical disease at the time, where the bulk is still small [25]. Patients with large tumors have the shortest time to imatinib failure. Abdominopelvic CT or MRI should be used as relapse usually occurs in peritoneum or liver. The same imaging technique should be used during follow-up of a certain patient. Physical examination and blood tests do not detect relapses which would otherwise be found by CT scans. Endoscopy is only indicated in familial GISTs and in some cases of R1 resections in gastric, esophageal, or rectal tumors. The recommendation for intermediate–high-risk localized resected patients is to perform a CT scan every 3–4 months in the first 3 years, then every 6 months up to 5 years and then annually. After stopping adjuvant imatinib, a closest follow-up is necessary for the next year [IV, D].

## References

[1] Poveda A, Rivera F, Martin J (2012). SEOM guidelines for gastrointestinal stromal sarcomas (GIST). Clin Transl Oncol Off Publ Fed Span Oncol Soc Natl Cancer Inst Mexico..

[2] Kalkmann J, Zeile M, Antoch G, Berger F, Diederich S, Dinter D (2012). Consensus report on the radiological management of patients with gastrointestinal stromal tumours (GIST): recommendations of the German GIST Imaging Working Group. Cancer Imaging Off Publ Int Cancer Imaging Soc.

[3] Bensimhon D, Soyer P, Boudiaf M, Fargeaudou Y, Nemeth J, Pocard M (2009). Imaging of gastrointestinal stromal tumors. J Radiol.

[4] Choi H, Charnsangavej C, Faria SC, Macapinlac HA, Burgess MA, Patel SR (2007). Correlation of computed tomography and positron emission tomography in patients with metastatic gastrointestinal stromal tumor treated at a single institution with imatinib mesylate: proposal of new computed tomography response criteria. J Clin Oncol Off J Am Soc Clin Oncol.

[5] Miettinen M, Lasota J, Sobin LH (2005). Gastrointestinal stromal tumors of the stomach in children and young adults: a clinicopathologic, immunohistochemical, and molecular genetic study of 44 cases with long-term follow-up and review of the literature. Am J Surg Pathol.

[6] Joensuu H, Vehtari A, Riihimaki J, Nishida T, Steigen SE, Brabec P (2012). Risk of recurrence of gastrointestinal stromal tumour after surgery: an analysis of pooled population-based cohorts. Lancet Oncol.

[7] Miettinen M, Lasota J (2006). Gastrointestinal stromal tumors: pathology and prognosis at different sites. Semin Diagn Pathol.

[8] Liegl B, Hornick JL, Corless CL, Fletcher CD (2009). Monoclonal antibody DOG1.1 shows higher sensitivity than KIT in the diagnosis of gastrointestinal stromal tumors, including unusual subtypes. Am J Surg Pathol.

[9] Serrano C, George S (2014). Recent advances in the treatment of gastrointestinal stromal tumors. Ther Adv Med Oncol..

[10] Corless CL, Barnett CM, Heinrich MC (2011). Gastrointestinal stromal tumours: origin and molecular oncology. Nat Rev Cancer.

[11] Corless CL, Schroeder A, Griffith D, Town A, McGreevey L, Harrell P (2005). PDGFRA mutations in gastrointestinal stromal tumors: frequency, spectrum and in vitro sensitivity to imatinib. J Clin Oncol.

[12] Wozniak A, Rutkowski P, Schoffski P, Ray-Coquard I, Hostein I, Schildhaus HU (2014). Tumor genotype is an independent prognostic factor in primary gastrointestinal stromal tumors of gastric origin: a European multicenter analysis based on ConticaGIST. Clin Cancer Res Off J Am Assoc Cancer Res..

[13] Heinrich MC, Corless CL, Demetri GD, Blanke CD, von Mehren M, Joensuu H (2003). Kinase mutations and imatinib response in patients with metastatic gastrointestinal stromal tumor. J Clin Oncol.

[14] Poveda A, del Muro XG, Lopez-Guerrero JA, Martinez V, Romero I, Valverde C (2014). GEIS 2013 guidelines for gastrointestinal sarcomas (GIST). Cancer Chemother Pharmacol.

[15] McCarter MD, Antonescu CR, Ballman KV, Maki RG, Pisters PW, Demetri GD (2012). Microscopically positive margins for primary gastrointestinal stromal tumors: analysis of risk factors and tumor recurrence. J Am Coll Surg.

[16] Casali PG, Le Cesne A, Poveda Velasco A, Kotasek D, Rutkowski P, Hohenberger P (2015). Time to Definitive Failure to the First Tyrosine Kinase Inhibitor in Localized GI Stromal Tumors Treated With Imatinib As an Adjuvant: a European Organisation for Research and Treatment of Cancer Soft Tissue and Bone Sarcoma Group Intergroup Randomized Trial in Collaboration With the Australasian Gastro-Intestinal Trials Group, UNICANCER, French Sarcoma Group, Italian Sarcoma Group, and Spanish Group for Research on Sarcomas. J Clin Oncol Off J Am Soc Clin Oncol..

[17] Joensuu H, Eriksson M, Sundby Hall K, Hartmann JT, Pink D, Schutte J (2012). One vs three years of adjuvant imatinib for operable gastrointestinal stromal tumor: a randomized trial. JAMA.

[18] Joensuu H, Eriksson M, Sundby Hall K, Reichardt A, Hartmann JT, Pink D (2016). Adjuvant imatinib for high-risk GI stromal tumor: analysis of a randomized trial. J Clin Oncol Off J Am Soc Clin Oncol..

[19] Blanke CD, Rankin C, Demetri GD, Ryan CW, von Mehren M, Benjamin RS (2008). Phase III randomized, intergroup trial assessing imatinib mesylate at two dose levels in patients with unresectable or metastatic gastrointestinal stromal tumors expressing the kit receptor tyrosine kinase: S0033. J Clin Oncol Off J Am Soc Clin Oncol..

[20] Verweij J, Casali PG, Zalcberg J, LeCesne A, Reichardt P, Blay JY (2004). Progression-free survival in gastrointestinal stromal tumours with high-dose imatinib: randomised trial. Lancet.

[21] Raut CP, Posner M, Desai J, Morgan JA, George S, Zahrieh D (2006). Surgical management of advanced gastrointestinal stromal tumors after treatment with targeted systemic therapy using kinase inhibitors. J Clin Oncol Off J Am Soc Clin Oncol..

[22] Blay JY, Le Cesne A, Ray-Coquard I, Bui B, Duffaud F, Delbaldo C (2007). Prospective multicentric randomized phase III study of imatinib in patients with advanced gastrointestinal stromal tumors comparing interruption vs continuation of treatment beyond 1 year: the French Sarcoma Group. J Clin Oncol Off J Am Soc Clin Oncol..

[23] Demetri GD, van Oosterom AT, Garrett CR, Blackstein ME, Shah MH, Verweij J (2006). Efficacy and safety of sunitinib in patients with advanced gastrointestinal stromal tumour after failure of imatinib: a randomised controlled trial. Lancet.

[24] Demetri GD, Reichardt P, Kang YK, Blay JY, Rutkowski P, Gelderblom H (2013). Efficacy and safety of regorafenib for advanced gastrointestinal stromal tumours after failure of imatinib and sunitinib (GRID): an international, multicentre, randomised, placebo-controlled, phase 3 trial. Lancet.

[25] Joensuu H, Martin-Broto J, Nishida T, Reichardt P, Schoffski P, Maki RG (2015). Follow-up strategies for patients with gastrointestinal stromal tumour treated with or without adjuvant imatinib after surgery. Eur J Cancer.

